# Are Reports of Psychological Stress Higher in Occupational Studies? A Systematic Review across Occupational and Population Based Studies

**DOI:** 10.1371/journal.pone.0078693

**Published:** 2013-11-04

**Authors:** Laura Goodwin, Ilan Ben-Zion, Nicola T. Fear, Matthew Hotopf, Stephen A. Stansfeld, Simon Wessely

**Affiliations:** 1 King's Centre for Military Health Research, Department of Psychological Medicine, Institute of Psychiatry, King's College London, London, United Kingdom; 2 Department of Psychological Medicine, Institute of Psychiatry, King's College London, London, United Kingdom; 3 Centre for Psychiatry, Queen Mary University of London, Barts and The London, London, United Kingdom; Academic Medical Center, The Netherlands

## Abstract

**Objectives:**

The general health questionnaire (GHQ) is commonly used to assess symptoms of common mental disorder (CMD). Prevalence estimates for CMD caseness from UK population studies are thought to be in the range of 14–17%, and the UK occupational studies of which we are aware indicate a higher prevalence. This review will synthesise the existing research using the GHQ from both population and occupational studies and will compare the weighted prevalence estimates between them.

**Methods:**

We conducted a systematic review and meta-analysis to examine the prevalence of CMD, as assessed by the GHQ, in all UK occupational and population studies conducted from 1990 onwards.

**Results:**

The search revealed 65 occupational papers which met the search criteria and 15 relevant papers for UK population studies. The weighted prevalence estimate for CMD across all occupational studies which used the same version and cut-off for the GHQ was 29.6% (95% confidence intervals (CIs) 27.3–31.9%) and for comparable population studies was significantly lower at 19.1% (95% CIs 17.3–20.8%). This difference was reduced after restricting the studies by response rate and sampling method (23.9% (95% CIs 20.5%–27.4%) vs. 19.2% (95 CIs 17.1%–21.3%)).

**Conclusions:**

Counter intuitively, the prevalence of CMD is higher in occupational studies, compared to population studies (which include individuals not in employment), although this difference narrowed after accounting for measures of study quality, including response rate and sampling method. This finding is inconsistent with the healthy worker effect, which would presume lower levels of psychological symptoms in individuals in employment. One explanation is that the GHQ is sensitive to contextual factors, and it seems possible that symptoms of CMD are over reported when participants know that they have been recruited to a study on the basis that they belong to a specific occupational group, as in nearly all “stress” surveys.

## Introduction

The general health questionnaire (GHQ) is one of the most commonly used measures to assess symptoms of common mental disorder (CMD) in the UK [1]. It has been administered in population studies (e.g. British Household Panel Survey) and more commonly in smaller studies of particular groups, such as occupational studies (e.g. a study of UK doctors [2]) to estimate the level of CMD in a specific population. It is generally believed that there are particular occupational groups who are exposed to a higher level of stress than other occupations, e.g. police officers and military personnel; however, there are few studies comparing rates of CMD across these occupations or to the general population.

UK prevalence estimates for common mental disorder from population studies are in the range of 14–17% [3], [4], with the prevalence of CMD in occupational studies, such as military personnel and London civil servants higher at 20% and 27% respectively [5], [6]. This difference is inconsistent with the ‘healthy worker effect’ and the assumption that healthier individuals are more likely to be selected into work, which is well established [7]. Furthermore, it is conflicting with the fact that there are many aspects of working which have a positive impact on mental health [8]. Occupational samples are also less likely to include the disabled, those with long term physical or mental health disorders, and by definition exclude the unemployed which in itself is a strong risk factor for poor mental health [9].

We have therefore undertaken a systematic review to identify UK studies that use the GHQ in either a population or an occupational setting and to examine whether a comparison between these types of surveys reveals a difference in the level of CMD caseness reported. The specific aim is to examine the prevalence of CMD caseness in all UK occupational and population studies, conducted from 1990 onwards, which have administered the GHQ, and to compare the weighted prevalence estimates between them.

## Methods

### Search strategy

The literature search was conducted in November 2011 using Medline, EMBASE and PsycInfo electronic databases to identify UK studies which had used the GHQ, covering the period from January 1990 to the date of the search. The search was restricted to UK studies due to the high quantity of international studies which have used the GHQ and also due to between country differences in occupational issues. The search terms were used as free text terms and were combined with Boolean operators. The initial search using the terms (*general health questionnaire* OR *GHQ*) was combined with (*United Kingdom* OR *UK OR Britain* OR *England* OR *Wales* OR *Scotland* OR *Ireland* OR *Northern Ireland* OR *British Isles*) using the AND operator.

### Inclusion criteria

#### Inclusion criteria for occupational and population studies

1. The study should have administered the General Health Questionnaire
2. The prevalence of CMD in the sample should be reported or available from the authors
3. Conducted in the UK
4. Sample size should be at least 100 participants
5. Sample should not include children, adolescents (<18years) or student groups
6. Should not use data from participants recruited in primary/secondary care, from GP records or who were recruited from a health care register

#### Inclusion criteria specific to occupational studies

1. The sample should be a particular occupational group(s)
2. Studies on individuals in a trainee position in a particular occupation (e.g. clinical psychology trainees) were included, but other types of student groups (e.g. medical undergraduates) were excluded

#### Inclusion criteria specific to population studies

1. Should include at least 1000 participants (higher than for occupational studies because the sampling frame for population studies would be expected to be larger)
2. Should not be a study of older adults (>60 y)

### Data extraction and analysis

Data were independently extracted by two researchers (LG & IBZ). Data from 20 occupational and 10 population studies were extracted by both researchers and agreement between the researchers was high. The data extracted from the articles were: author, title and date of publication; information about the population and the location of the study; study design and type of sampling; number of participants and response rate; sample characteristics (e.g. gender split and age); which version of the GHQ questionnaire was administered and the cut-off used; and data on the prevalence of CMD. The numerator (the number of CMD cases) and the denominator (the sample size or number of participants who had data on CMD) were entered into the review database so that the prevalence (%) of CMD, standard errors and 95% confidence intervals (CI) could be calculated. Stata v11.0 was used for all data analyses [10] and meta analyses were conducted to produce weighted estimates and to examine the between study heterogeneity.

1. The metan command was used to produce the forest plots, displaying the prevalence of CMD and 95% CIs for each sample, grouped by occupation for the occupational studies. Forest plots were produced across all of the occupational studies, displaying the weighted estimates by occupational group and overall across all of the studies. A further forest plot displayed the weighted estimates across the occupational studies and for the population studies, so that the estimates could be compared between these categories.
2. To further investigate the issue of heterogeneity, and the difference between the two types of study, analyses were conducted which restricted the studies included to those which used the GHQ-12 version of the questionnaire and with a cut-off of 3/4 (41 occupational and 19 population studies) so that the prevalence estimates between studies should be more comparable. Random effects meta-analysis models were conducted within these restricted studies.
3. Meta regressions were conducted using the metareg command to examine whether response rate (categorised as less than 50% vs. equal to or more than 50%) and sampling method (split into studies which used random sampling or aimed to recruit all participants from the sampling frame vs. studies which used non-random sampling) were associated with the prevalence of CMD. These variables were entered individually into meta regressions for the occupational and population studies separately.
4. A further meta-analysis was conducted which restricted the studies to those with used the same version and cut-off of the GHQ, which was also restricted to studies which had a response rate of at least 50% and which used random sampling and a forest plot was produced displaying the weighted estimates for the occupational and the population studies.

### Ethical approval

Ethical approval was not required for this systematic review.

## Results

### Study selection

The initial search terms *general health questionnaire* OR *GHQ* (restricted to abstracts in the English language and to the period from January 1990 – current) identified **10106** abstracts. A further search using the terms: *United Kingdom* OR *UK OR Britain* OR *England* OR *Wales* OR *Scotland* OR *Ireland* OR *Northern Ireland* OR *British Isles* was combined with the initial search using the AND operator, to restrict the search only to UK based studies. This reduced the number of abstracts to **1458** that met the initial search criteria: 494 of these abstracts were in Medline, 510 in EMBASE and 454 in PsycINFO. Examination of the titles revealed that 502 abstracts from the three databases were duplicates, so there were **956** articles that met the search criteria for this review.

**Figure 1:**
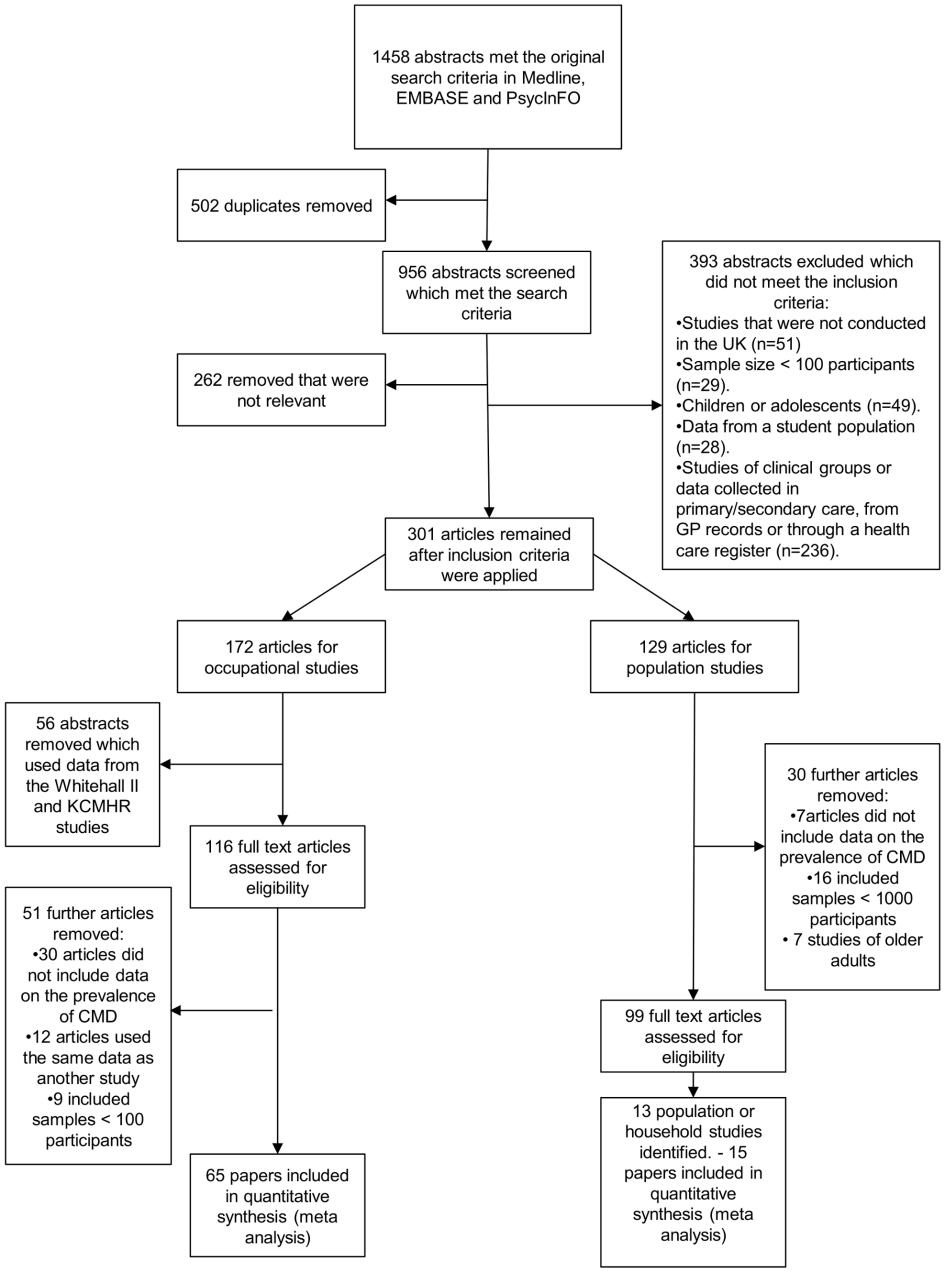
PRISMA flow diagram showing the search process and selection of relevant abstracts.

Initial screening of the 956 abstracts showed that 262 of these were not relevant to the review, or were literature reviews. Further screening of the 694 remaining abstracts excluded a further 393 abstracts on the basis of the overall inclusion criteria with 301 abstracts remaining (see figure 1).

### Selection of occupational studies

**172** of these 301 abstracts were found to be occupational studies (see figure 1). Of these, 22 articles used data from the Whitehall II data and only the paper including data from the first wave was included, so a further 21 articles were excluded. 38 abstracts used data from the King's Centre for Military Health (KCMHR) military studies, of which 3 of the main studies were included in the review as the majority of the remaining papers used data from these same studies (or from a different phase of the same study). Therefore 56 papers were removed, which used these datasets, before the full articles were collated.

The **116** remaining articles were sent to full review, however, 27 did not provide data on the prevalence of CMD and this information was not available from the authors. Further reasons that papers were not included in the review included: 12 papers used the same data as another paper included in the review, 9 included samples <100 participants (this information was not apparent from the abstract), 3 papers did not collect data on CMD. **65** occupational papers were included in the final review for which data on CMD were available.

### Selection of population studies

**129** of the 301 abstracts were found to be population or household studies and the full articles were collated which were sent to review (see figure 1). Out of these, 16 studies did not include at least 1000 participants, 7 studies were of older adults and 7 were excluded which either did not administer the GHQ or the prevalence of CMD was not available.

Within the 99 remaining studies, there were **13 population based studies** which were eligible for inclusion in the review and some of the population studies were reported in multiple abstracts (e.g. 38 used data from the British Household Panel Survey). There was also 5 out of the 99 studies that reported data from more than one population study.

There were 11 papers identified within the original search that included the relevant data on the prevalence of CMD for either one, or in some cases two, of the population studies (due to some papers reporting data from multiple population studies). The decision was made to report data from only one time-point/assessment for all of the longitudinal or repeated cross-sectional studies. Data was not available in the 11 identified papers for the Health Survey for England (data was extracted from an official published report from the NHS Information Centre), for the National Child Development Study (this data was extracted from the dataset by the researcher), for the Scottish Health Survey (extracted from an official report), nor for the West of Scotland Twenty-07 study (data extracted for the 3 age cohorts by the Twenty-07 data manager).

Data was therefore reported on **13 population studies**: *Aberdeen Children of the 1950s study*; *British Household Panel Survey* wave 1; *English and Welsh Civil and Social Justice Survey*; *Health and Lifestyle Survey*; *Health Survey for England* 1995; *MRC National Health and Development Study (GHQ administered at 53 y)*; *National Child Development Study (GHQ administered at 42 y)*; *Northern Ireland Health and Wellbeing Survey*; *Northern Ireland Household Panel Survey*; *Renfrew and Paisley (MIDSPAN) study*; *Scottish Health Survey* 1995 wave; *West of London Survey; West of Scotland Twenty-07 Study* [which included 3 age cohorts: 1930s cohort, 1950s cohort, and 1970s cohort].

### Occupational studies

#### Study characteristics

Out of the 65 studies identified within this review (table 1), the majority had been conducted in health professionals and NHS staff (n = 41). Five had been carried out in military personnel, four in white collar workers, five in academics and teachers and the remaining 10 studies in social services staff, police, manual workers and chaplains. 47 studies recruited sample sizes ranging from 100 to 1000 participants; however, there were 18 studies which recruited a large sample, including a number of studies of NHS staff (e.g. a study of 1308 UK hospital consultants [11]), the Whitehall II study of civil servants (>10 000 staff from 20 London based civil service departments [6]) and studies of military personnel (e.g. a cross sectional survey of >10 000 UK armed forces personnel [5]). Twenty one studies used random sampling (or invited all participants in the sampling frame to participate in the study) and 44 used non-random sampling. Forty four studies had a response rate of at least 50%, 17 studies reported a response rate of less than 50% and 4 did not report this information.

**Table 1:** Data extraction table for occupation studies (ordered by occupational group).

Occupational group	First author (year of publication) and title	Population and location	Type of study and sampling strategy	No. of participants (no. who completed GHQ if different)	Response rate	Sample characteristics	Which version of the GHQ questionnaire was used (Cut-off used)	Prevalence CMD in full sample (%, 95% CI)
**Academics and teachers**	Emslie et al. (2002) Gender differences in mental health: evidence from 3 organisations[69]	White collar workers from a bank, a university and the civil service in the UK (Only the data from the British university is presented as the other data is presented in another study in the review).	Cross-sectional questionnaire study, questionnaires distributed to all university employees in white collar occupations.	1641	67%	1009 men, 632 women. Mean ages: males – 44 y, females – 39 y	GHQ-12 (2/3)	Males - 24%, Females - 27%
**Academics and teachers**	Kinman & Jones (2008a) Effort reward balance and over commitment:Predicting strain in academic employees in the UK[43]	UK academics	Cross sectional study. Random sampling.	844	1108/5000 staff (22%), but only 844 included in current study	59% male. 77% aged 40 y or over	GHQ-12 (3/4)	41.80%
**Academics and teachers**	Kinman (2008b) Work stressors, health and sense of coherence in UK academic employees[44]	UK academics	Cross-sectional postal study. Random sample of 1000 UK academic employees working full time.	465	47%	59% male. Mean 46 y (S.D. 7.4).	GHQ-12 (3/4)	43.40%
**Academics and teachers**	McClenahan et al. (2007) The importance of context specificity in work stress research: A test of the demand-control-support model in academics[45]	UK academics	Cross sectional and non-random (only lecturers and senior lecturers were included)	166	225 responded – (23%) – analyses restricted to 166 lecturers and senior lecturers	105 men and 61 women. Mean age 44 y (SD 10, range 26-64).	GHQ-12 (3/4)	31.80%
**Academics and teachers**	Miller & Travers (2005) Ethnicity and the experience of work: Job stress and satisfaction of minority ethnic teachers in the UK[46]	Minority ethnic teachers in the UK	Cross sectional. Non-random sampling.	208	208/1900 (9%)	77.7% female (160). Age range 21–65 y	GHQ-12 (3/4)	44.2% (Males - 34.8%, Females - 46.3%)
**Health professionals and NHS staff**	Alexander and Klein (2001) Ambulance personnel and critical incidents: Impact of accident and emergency work on mental health and emotional well-being[70]	Ambulance personnel, UK	Cross sectional survey, non-random sampling.	110	69%	86% male. Age range: 20–29 y: 17%, 30–39 y: 47%, 40–49 y: 22%, 50+y: 14%.	GHQ 28(4/5)	32%
**Health professionals and NHS staff**	Appleton et al, (1998) A survey of job satisfaction, sources of stress and psychological symptoms among general practitioners in Leeds[71]	GPs, Leeds, UK	Cross-sectional postal questionnaire, non-random sampling.	285	70%	61% male.Mean age - 42.2 y (SD: 9.21 y, range: 28–68 y)	GHQ-12(2/3)	52%
**Health professionals and NHS staff**	Baldwin et al. (1997) Young Doctors' Health- II. Health and Health Behaviour[72]	Junior Doctors, UK	Cross sectional study, non-random sampling.	142	95%	54.9% male. Mean age - 25 y	GHQ-28, (4/5)	37%
**Health professionals and NHS staff**	Bamber & McMahon (2008) Danger - early maladaptive schemas at work! [73]	NHS staff at a Primary Care Trust and Hospital Trust in York, England	Cross sectional survey. Non-random sampling.	249	24.80%	27% male. Mean age 42 y (S.D. 9.8 y, range 21–64 y)	GHQ-28 (4/5)	34.1%
**Health professionals and NHS staff**	Berman et al. (2007) Occupational stress in palliative medicine, medical oncology and clinical oncology specialist registrars[15]	Specialist registrars in palliative medicine, clinical oncology and medical oncology, UK	Cross-sectional mail survey, non-random sampling.	449 (390)	65.70%	34.2% male, Mean ages (SD): Overall 32.7 y (3.3), Palliative medicine 33 y (4.1), Medical oncology 32.9 y (2.9), Clinical oncology 32.3 y (3.0),	GHQ-12 (3/4)	26.2%. For individual groups. Palliative: 19.5%, Medical 29.1%, Clinical 28.7%.
**Health professionals and NHS staff**	Burbeck et al. (2002) Occupational stress in consultants in accident and emergency medicine: a national survey of levels of stress at work[16]	Accident and emergency consultants, UK	Cross sectional postal survey, non-random sampling.	350	78%	83.4% male. Median age: 45 y, range 31–67.	GHQ-12(3/4)	44%
**Health professionals and NHS staff**	Butterworth et al. (1999) Stress, coping, burnout and job satisfaction in British nurses: Findings from the clinical supervision evaluation project[74]	Nurses, UK	Cross-sectional survey, non-random sampling.	586	96%	10% male. Age range: 21–30 y: 28%, 31–40 y: 37%, 41–50 y: 25%, 50+y: 11%.	GHQ-28 (4/5)	Overall: 30%, ward staff: 28%, community nurses 41%, social work trainees 64%.
**Health professionals and NHS staff**	Calnan et al. (2001) Mental health and stress in the workplace: the case of general practice in the UK[17]	GP practice staff, UK	Cross sectional survey, random sample of general practices and non-random within practices.	719	70%	Reported to be mainly female and in the age range 35–44 y.	GHQ-12(3/4)	Overall 23%. Doctors and nurses 30%, district nurses 27%, health visitors 24%, practice nurses 22%, receptionists 17%, admin/clerical staff 19%.
**Health professionals and NHS staff**	Caplan et al. (1994) Stress, anxiety and depression in hospital consultants, general practitioners and senior health service managers[75]	Hospital consultants, GPs and health services managers, Lincoln, England	Cross sectional survey. Non-random sampling.	389 (65 consultants, 257 GPs, 67 managers)	80% consultants, 80% GPs, 56% managers	Not reported	GHQ-28 (5/6)	Overall - 46%. Consultants - 46%, GPs - 48%, Managers - 46%
**Health professionals and NHS staff**	Catt et al. (2005) The informational roles and psychological health of members of 10 oncology multidisciplinary teams in the UK[18]	Members of multidisciplinary cancer teams in England, Wales & Scotland	Cross sectional survey. Non-random sampling.	144 (142)	Not reported	Not reported	GHQ-12 (3/4)	25/142 (18%)
**Health professionals and NHS staff**	Coomber et al. (2002) Stress in UK intensive care unit doctors[19]	ICU doctors in the UK	Cross sectional postal survey. Non-random sampling.	627 (610)	70% total response (627/896) or 80% of potentially eligible responders (627/788)	3.4% male. Mean age 41.8 y (S.D. 7.3 y)	GHQ-12 (3/4)	28.5% (24.9-32.1%) (Males - 28.3%, Females - 29.3%)
**Health professionals and NHS staff**	Cushway (1992) Stress in clinical psychology trainees[76]	Clinical psychology trainees in the UK	Cross sectional survey. Non-random sampling.	287	76%	27% male. Mean age 27.4 y (S.D. 4.9 y) range 22–42 y	GHQ-28 (4/5)	59%
**Health professionals and NHS staff**	Cushway and Tyler (1994) Stress and coping in Clinical Psychologists[77]	Clinical Psychologists, West Midlands UK	Cross-sectional, Non-random sampling.	101 (95)	67%	38.4% male. Mean age: 37.6 y (S.D. 7.1 y) range: 25–61 y.	GHQ-28(4/5)	29.40%
**Health professionals and NHS staff**	Edwards et al. (2000) Stressors, moderators and stress outcomes: findings from the All-Wales Community Mental Health Nurse Study [78]	Community mental health nurses, UK	Cross sectional survey, non-random sampling.	301	49%	38% male. Mean age: 40.4 y (S.D.7.2 y), range 23–63 y.	GHQ-12(1/2)	35%
**Health professionals and NHS staff**	Fagin et al. (1995) The Claybury community psychiatric nurse study[79]	Community psychiatric nurses. North East Thames region, UK.	Cross sectional survey. Non-random sampling.	250 community psychiatric nurses, 323 ward-based psychiatric nurses (WBPN)	Not reported	CPN - 38% male, WBPNs - 37.2% male. Mean age: CPN - 38.9 y, WBPN - 34.7 y	GHQ-28 (4/5)	CPNs - 41%, WBPNs - 27.9%
**Health professionals and NHS staff**	Glozier et al. (2006) Attitudes of nursing staff towards co-workers returning from psychiatric and physical illnesses[80]	Nursing staff in one UK NHS Trust	Cross sectional survey. Random sampling.	117 (103)	22%	8.5% male. Mean ages for the 3 groups ranged from 35.6 y (S.D. 9.4 y) to 38.6 y (S.D. 8.6 y)	GHQ-12 (2/3)	27.60%
**Health professionals and NHS staff**	Gorter et al. (2011) Burnout and engagement in relation with job demands and resources among dental staff in Northern Ireland[21]	Dental staff, Northern Ireland, UK	Cross-sectional mail survey, non-random sampling.	135	45%	25.2% male. Age: 50% aged 40–55 y.	GHQ-12 (3/4)	25%
**Health professionals and NHS staff**	Guthrie et al. (1999) Sources of stress, psychological distress and burnout in psychiatrists[22]	Psychiatrists, Manchester, UK	Cross-sectional survey, non-random sampling.	106	76.80%	51.1% male. Consultants (59.6% male), Senior registrars (60.7% male), Registrars/SHOs (35.5% male)	GHQ-12(3/4)	31.40%
**Health professionals and NHS staff**	Hardy et al. (1999) Validation of the General Health Questionnaire-12 Using a Sample of Employees From England's Health Care Services[23]	NHS Staff, UK	Cross-sectional interview, non-random sampling.	11 637	Estimated response rate between 61–65%	Mean age:38 y, SD 10.3 y, Range: 20–62 y	GHQ -12(3/4)	27%
**Health professionals and NHS staff**	Hughes & Parkes (2007) Work hours and well-being: The roles of work-time control and work-family interference[24]	Two UK public sector organisations	Cross sectional study. Random sampling for primary care sample and non-random for local government.	292	44% primary care and 46% local government	All females. Mean age 42 y (S.D. 10 y, range 19–66 y).	GHQ-12 (3/4)	Primary care - 52/212 (24.5%), Local government office - 23/80 (28.8%)
**Health professionals and NHS staff**	Kumary & Baker (2008) Stresses reported by UK trainee counselling psychologists[25]	Counselling psychology trainees, UK	Cross sectional postal survey. Non-random sampling.	109 (93)	41%	19% male. Age range: 20–30 y – 47 (43%), 31–40 y - 36 (34%), 41 y+ - 24 (22%)	GHQ-12 (3/4)	58%
**Health professionals and NHS staff**	Loretto et al. (2010) Workplace change and employee mental health: results from a longitudinal study[26]	NHS staff in 6 UK NHS Trusts.	First phase of a longitudinal postal survey. Stratified random sampling.	5385	18.40%	4/5 female. Mean age: 41 y (S.D. 10 y, range 17–70 y).	GHQ-12 (3/4)	24.20%
**Health professionals and NHS staff**	Macpherson et al. (1994) Psychological distress among workers caring for the elderly[81]	Workers caring for the elderly, UK	Cross-sectional, Non-random sampling.	188	67.40%	9% male. Mean age: 38 y (S.D. 11.85 y, range 16–69 y)	GHQ-30, CGHQ scoring system (12/13)	26.6% (males 35.3%, females 25.7%)
**Health professionals and NHS staff**	McKinstry et al. (2004) The MAGPI (Morale Assessment in General Practice Index): a new way for doctors to self-assess their morale[82]	GPs in South East England, UK	Cross sectional survey. Non-random sampling.	613	70%	Not reported	GHQ-28 (4/5)	31%
**Health professionals and NHS staff**	McManus et al. (1999) Are UK doctors particularly stressed?[2]	Doctors, UK	Cross-sectional survey, random sampling within the medical directory.	1261 (1013)	81%	Not reported	GHQ-12(3/4)	16.90%
**Health professionals and NHS staff**	McManus et al. (2000) Duties of a doctor: UK doctors and Good Medical Practice[27]	Doctors, UK	Cross sectional questionnaire study, representative stratified sampling.	556 (448)	73%	Not reported	GHQ-12 (3/4)	15%
**Health professionals and NHS staff**	McManus et al. (2002) How consultants, hospitals, trusts and deaneries affect pre-registration house officer posts: a multilevel model[28]	Pre-registration house officers (PRHOs), UK	Cross-sectional, Non-random sampling.	1435 (1330)	58.40%	Not reported	GHQ-12(3/4)	31.70%
**Health professionals and NHS staff**	McManus et al. (2003) A levels and intelligence as predictors of medical careers in UK doctors - 20 yr prospective study[29]	Doctors in the UK - originally recruited in London	Phase 2 of a prospective study of clinical students. Non random sampling.	349	464 out of the original 511 were on the 2001 Medical Register and 349 responded (73% response)	Not reported	GHQ-12 (3/4)	18%
**Health professionals and NHS staff**	McManus et al. (2004) Stress, burnout and doctors' attitudes to work are determined by personality and learning style - A 12 yr longitudinal study of medical graduates[30]	Doctors who had previously applied to one of 5 medical schools as a student in the UK	Cross sectional assessment in a prospective study. Non-random sampling.	1668 (1617)	2635 doctors were applicable for this study and 1668 of these responded (63.3%)	Mean age 30.4 y (S.D. 1.86 y) range 28.3–49.2 y	GHQ-12 (3/4)	345/1617 (21.3%)
**Health professionals and NHS staff**	Oyefeso et al. (2008) Prevalence and associated factors in burnout and psychological morbidity among substance misuse professionals[14]	Substance misuse professionals, South Thames region, England, UK	Cross-sectional study, non-random sampling.	194 (187)	69%	43% male. Age: Mean (SD): 38 (9.9)	GHQ-12(3/4)	82.3%.82.40% males, 82.2% females.
**Health professionals and NHS staff**	Paice et al. (2002) Stressful incidents, stress and coping strategies in the pre-registration house officer year[31]	PRHOs - Newly qualified doctors, Hospitals, UK	Cross sectional postal study, non-random sampling.	1435 (1430)	58.40%	45.2% male.	GHQ-12 (3/4)	31.30%
**Health professionals and NHS staff**	Patterson & Bell (2000) Supporting staff in employment: the emotional wellbeing of staff in an NHS psychiatric hospital[83]	NHS staff of a large psychiatric service in Scotland, UK	Cross sectional study. Non-random sampling.	287	47.90%	Not reported	GHQ-28 (4/5)	32.90%
**Health professionals and NHS staff**	Ramirez et al. (1995) Burnout and psychiatric disorder among cancer clinicians [32]	Consultant non-surgical oncologists, UK	Cross sectional study. Non-random sampling.	393 (60 medical oncologists, 207 clinical oncologists, 126 palliative care specialists) (392)	83% (87% medical oncologists, 82% clinical oncologists, 82% palliative care specialists)	Overall: 74% male. Medical oncologists -92% male, clinical oncologists – 79% male, palliative care specialists – 58% male.Age range - < = 35 y - 5%, 36–45 y - 49%, 46–55 y - 30%, >55 y - 16%.	GHQ-12 (3/4)	Overall - 28% (Medical oncologists - 32%, clinical oncologists 28%, palliative care specialists 25%)
**Health professionals and NHS staff**	Ramirez et al. (1996) Mental health of hospital consultants: the effects of stress and satisfaction at work[33]	Hospital consultants, UK	Cross-sectional questionnaire based study, random sampling.	882	78%	88% male. Age ranges - <35 y - 3%, 36–45 y - 44%, 46–55 y - 37%, >55 y - 16%.	GHQ-12 (3/4)	27%
**Health professionals and NHS staff**	Sharma et al, (2008a) Stress and burnout among colorectal surgeons and colorectal nurse specialists working in the National Health Service[34]	Colorectal surgeons and nurses, Ireland and rest of UK	Cross sectional, non-random sampling	253 surgeons, 177 nurses (251 surgeons, 176 nurses)	Surgeons: 55.6%, Nurses: 54.3%	Surgeons: 90% male. Nurses: 4.5% male. Mean age: Nurses: 42.8 y, Surgeons: 47.7 y	GHQ-12(3/4)	Surgeons 30.2%, nurses 30.3%
**Health professionals and NHS staff**	Sharma et al. (2008b) Stress and burnout in colorectal and vascular surgical consultants working in the UK National Health Service[35]	Vascular surgeons, UK (Data for colorectal surgeons reported in another paper)	Cross-sectional survey, non-random sampling	248(244)	62.3%	94.3% male. Mean age 47.1 y (range 31–65 y)	GHQ-12(3/4)	Vascular surgeons: 35.7%
**Health professionals and NHS staff**	Sheikh & Hurwitz (2000) Psychological morbidity in general practice: a descriptive and explanatory study[84]	General practice managers from two health authorities in Southern England, UK	Cross sectional postal survey. Non-random sampling.	111	74.50%	87% female. Mean age 46.5 y (S.D. 9.1)	GHQ-28 (5/6)	41/111 (37%)
**Health professionals and NHS staff**	Taylor et al. (2005) Changes in mental health of UK hospital consultants since the mid-1990s[11]	Hospital consultants in the UK	Cross sectional survey. 2002 follow-up. Non-random.	1308	1308/1794 (73%)	81% female	GHQ-12 (3/4)	32%
**Health professionals and NHS staff**	Tyler et al. (1991) Stress and well-being in nurses: a comparison of the public and private sectors[85]	Nurses from 4 NHS and 3 private hospitals in the Midlands, England, UK	Cross sectional survey. Non-random sampling.	156	Overall 57%, 53% for the public sector, 63% for private	Not reported	GHQ-28 (3/4)	33%
**Health professionals and NHS staff**	Wall et al. (1997) Minor psychiatric disorder in NHS trust staff: occupational and gender differences[36]	Employees from 19 NHS trusts in England, UK	Cross sectional survey. Participants from larger occupational groups were randomly sampled and from smaller groups were non-randomly sampled.	11637 (11291)	61–65%	26% male	GHQ-12 (3/4)	26.80%
**Health professionals and NHS staff**	Wray et al. (2009) A wealth of knowledge: A survey of the employment experiences of older nurses and midwives in the NHS[86]	Nurses and midwives in NHS and primary care trusts in UK.	Cross sectional postal survey. All qualified nurses and midwives over 50 y of age and a random sample of 20% of those aged <50 y.	510	20%	10% male. 62.1% were aged 50 y and over.	GHQ-12 (23/24 Likert scoring)	41%
**Manual workers**	Avery et al. (1998) Mental and physical health of miners following the 1992 national pit closure programme[87]	Males working in the mining industry in 1992, Nottinghamshire, England, UK	Cross sectional. Non-random sampling.	241 current miners (226)	51% overall	All males. 45.6% under 35 y, 44.8% 36–49 y and 8.7% 50 y and over	GHQ-12 (2/3)	104 (46%)
**Manual workers**	Booth and Lloyd (1999) Stress in Farmers[88]	Farmers, South-West of England, UK	Cross sectional postal survey, non-random sampling.	303	30.30%	89.4% male. 36% were aged between 30–39 y.	GHQ-28(4/5)	35%
**Manual workers**	Hussain (2004) Musculoskeletal symptoms among truck assembly workers[41]	Assembly workers at a UK based company	Cross sectional study. Non-random sampling.	323	323/461 (70%)	Mean age 36.5 y (S.D. 12.3 y)	GHQ-12 (3/4)	51/323 (15.8%)
**Manual workers**	Wadsworth et al. (2008) Fatigue and health in seafaring population[42]	Seafarers, UK	Cross-sectional questionnaire survey, non-random sampling.	1855 (1809)	20%	96% male. Mean age 43.5 y, median 45 y, range 17–66 y.	GHQ-12 (3/4)	18%
**Military personnel**	Bridger et al. (2007) Occupational stress and strain in the naval service:1999 and 2004[37]	Personnel in Royal Navy and Royal Marines in the UK	Cross sectional assessment in 1999. Stratified random sampling.	1707	78%,	1217 males, 490 females, Mean age - 31.05 y (S.D. 7.63 y),	GHQ-12 (3/4)	32%, (Males-31%, Females-43%,)
**Military personnel**	Bridger et al. (2008) Occupational stress and strain in the Royal Navy 2007[38]	Personnel in Royal Navy and Royal Marines in the UK	Phase 1 of a longitudinal study conducted from 2007–8. Stratified sample of 5000 naval personnel	2596	2596/4542 (57%)	Mean age 34.7 y	GHQ-12 (3/4)	31.5% (Males - 27.8% (26-30%), Females - 37.3% (34-50%)
**Military personnel**	Hotopf et al. (2006) The health of UK military personnel who deployed to the 2003 Iraq war: a cohort study[5]	UK armed forces personnel	Cross sectional assessment. First phase of a cohort study. Random stratified sampling.	10272 (Era group - 5550, TELIC - 4722)	58.7%	Era -90% male, TELIC - 92% male.	GHQ-12 (3/4)	Era - 20% (1071/5481), TELIC - 20% (953/4631)
**Military personnel**	Jones et al. (2006) The burden of psychological symptoms in UK armed forces[39]	UK armed forces personnel	Cross sectional postal survey. Random sampling.	1382	65%	92% male, Mean age 32 y (S.D. 7.9 y)	GHQ-12 (3/4)	270/1382 (20%)
**Military personnel**	Unwin et al. (1999) Health of UK servicemen who served in Persian Gulf War[89]	UK armed forces personnel	Cross sectional postal survey. Random stratified sampling.	8195 (7507)	65%	All males. Mean age 34.7 years	GHQ-12 (2/3)	Overall - 31.2% (Gulf - 39.2%, Bosnia - 26.3%, Era - 24.0%)
**Other**	Lloyd-Williams et al. (2004) A prospective study of the roles, responsibilities and stresses of chaplains working within a hospice[48]	Chaplains working within a hospice, UK	Cross-sectional study. Questionnaires sent to all chaplains working in a hospice in the UK.	115	57%	Not reported	GHQ-12(3/4)	24%
**Police**	Biggam et al. (1997) Coping with the occupational stressors of police work[90]	Serving Scottish police officers, UK	Cross sectional study. Non-random sampling.	699	Not reported	87.5% male. Mean age 35.9 y (range 18–56)	GHQ-28 (4/5)	22.8% (n = 160)
**Police**	Brown et al. (1999) Distinguishing traumatic, vicarious and routine operational stressor exposure and attendant adverse consequences in a sample of police officers[91]	Police Officers, UK	Cross-sectional study. Non-random sampling.	593	60% female, 61% male.	61.9% male. Mean age: males - 35.1 y (8.09 y), females - 29.4 y (6.0 y)	GHQ-12 (1/2)	40%
**Social services staff**	Coffey et al. (2004) Stress in Social Services: Mental Well-being, Constraints and Job Satisfaction[40]	Social Services staff, North-West England, UK	Cross-sectional study. Non-random sampling.	1234 (1,078)	32.70%	19% male. Age range: 50+y: -29%	GHQ-12 (3/4)	36%
**Social services staff**	Evans et al. (2006) Mental health, burnout and job satisfaction among mental health social workers in England and Wales[20]	Mental health social workers in England & Wales, UK	Cross sectional postal study. Random sampling.	237	237/610 (39%) questionnaires received out of those distributed. Adjusted response rate of 49% as 125 participants were not eligible for the study	39% male. Mean age 46 y (9.2 y)	GHQ-12 (3/4)	111 (47%)
**Social services staff**	Kinman & Grant (2011) Exploring stress resilience in trainee social workers[92]	Trainee social workers in the UK	Cross sectional study. Non-random sampling.	240	Not reported	18% male. Mean age 33.7 y (S.D. 9.04 y)	GHQ-12 (2/3)	43%
**White collar workers**	Bond & Donaldson-Feilder (2004) The relative importance of psychological acceptance and emotional intelligence to workplace well-being[13]	5 organisations: a manufacturing company, the London office of an overseas government, the management consultancy arm of a large accountancy firm, the corporate headquarters of an insurance broker, and a financial services consultancy, UK	Cross-sectional study. Non-random sampling.	290	51%	Male: 51%. Age: Mean: 38.19 y, SD: 10.55 y	GHQ-12(3/4)	14.40%
**White collar workers**	Emslie et al. (1999) Problematizing gender, work and health: the relationship between gender, occupational grade, working conditions and minor morbidity in full-time bank employees[93]	Full time bank employees, Scotland, UK	Cross sectional survey, Random sample other than for particular occupational grades for which all individuals invited to participate.	2176 (2130)	76%	51.1% male. Mean age: 35.6 y, 60% aged 35 y or younger	GHQ-12(2/3)	Overall: 26.6%, (males: 25.0%, females: 28.0%). Clerical: 25%, supervisors: 27.6%, managers: 29.1%
**White collar workers**	Guppy and Weatherstone (1997) Coping strategies, dysfunctional attitudes and psychological well-being in white collar public sector employees[47]	Public sector employees, UK	Cross-sectional, random sample of offices in which all individuals invited to participate.	274	99.60%	31% male. Mean age 33 y, (S.D. 9.6 y, range: 17–59 y)	GHQ-12 (3/4)	26.30%
**White collar workers**	Stansfeld et al. (1992) Social class and minor psychiatric disorder in British Civil Servants: a validated screening survey using the General Health Questionnaire [94]	Staff from 20 London based civil service departments aged between 35–55 years, UK.	First phase of a longitudinal prospective study (1985–8). All civil servants in the selected departments, aged 33–55 years, invited to participate.	10314 (10195)	73%	67% male. Aged 35–55 y.	GHQ-30 (4/5)	26.9%

#### How was common mental disorder measured?

The version of the GHQ that was administered varied between studies, in addition to the cut-off that was utilised. 49 assessed CMD using the GHQ-12, 14 used the GHQ-28, and 2 used the GHQ-30. 39 out of the 49 studies that administered the GHQ-12 used a cut-off of 3/4 as the criteria for being a case of CMD and 11 out of the 14 studies which used the GHQ-28 used a cut-off of 4/5, suggesting that these were the most common criteria for occupational studies which assess CMD.

#### Prevalence of common mental disorder

**Figure 2:**
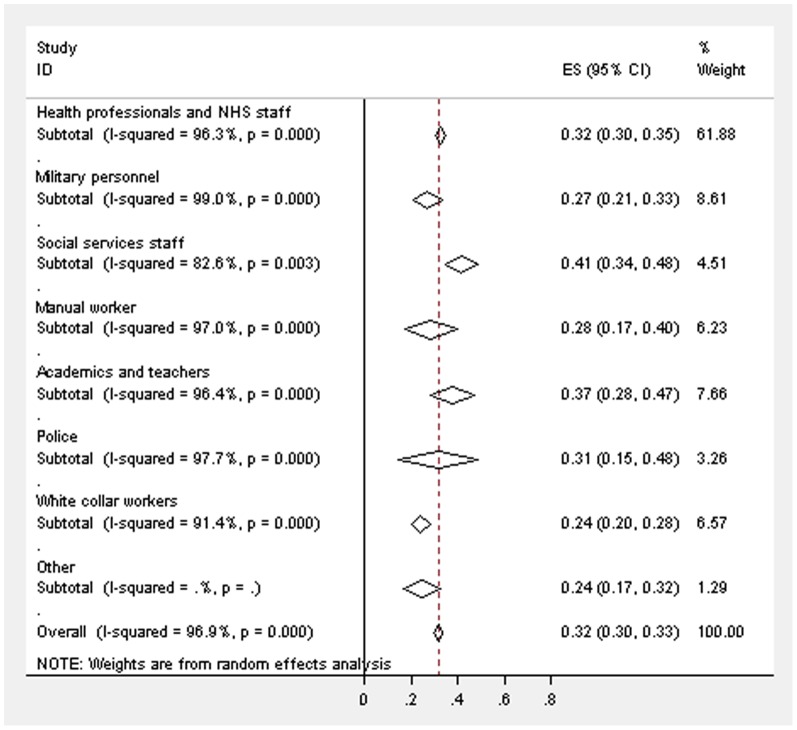
Forest plot displaying the weighted estimates for CMD caseness across the occupational groups.

Figure 2 displays the weighted estimates for CMD caseness across the different occupational groups, with an overall estimate of 31.6% (95% confidence intervals (CIs) 29.9–33.4%). There was evidence for high heterogeneity [12] from random effect models between the studies in all of the occupational groups (academics and teachers - I^2^ = 96.4%; health professionals and NHS staff - I^2^ = 96.3%; manual workers - I^2^ = 97.0%; military personnel - I^2^ = 99.0%; police - I^2^ = 97.7%; social services staff - I^2^ = 82.6%; white collar workers - I^2^ = 91.4%). The prevalence of CMD appears to be highest within the studies of academics/teachers (4 out of the 5 studies included academics working at a university) and social services staff, but the differences between the occupational groups were not statistically significant as the CIs overlapped. The weighted estimate for the prevalence of CMD in studies of academics and teachers was 37.2% (95% CIs 27.8–46.7%), in health professionals and NHS staff was 32.4% (95% CIs 30.0–34.7%), in manual workers was 28.4% (95% CIs 17.0–39.7%), in military personnel was 26.8% (95% CIs 20.7–33.0%), a prevalence of 24.3% (95% CIs 16.5–32.2%) in one study of chaplains, 31.4% (95% CIs 14.6–48.1%) in the police, 41.5% (95% CIs 34.4–48.5%) in social services staff and 23.9% (95% CIs 20.0–27.8%) in white collar workers. The lowest prevalence of CMD for an individual study was 14.5% in a study of white collar workers from 5 different organisations [13], with the highest in a study of substance misuse professionals of 82.4% [14].

#### Occupational studies which used the GHQ-12 (cut-off 3/4)

**Figure 3:**
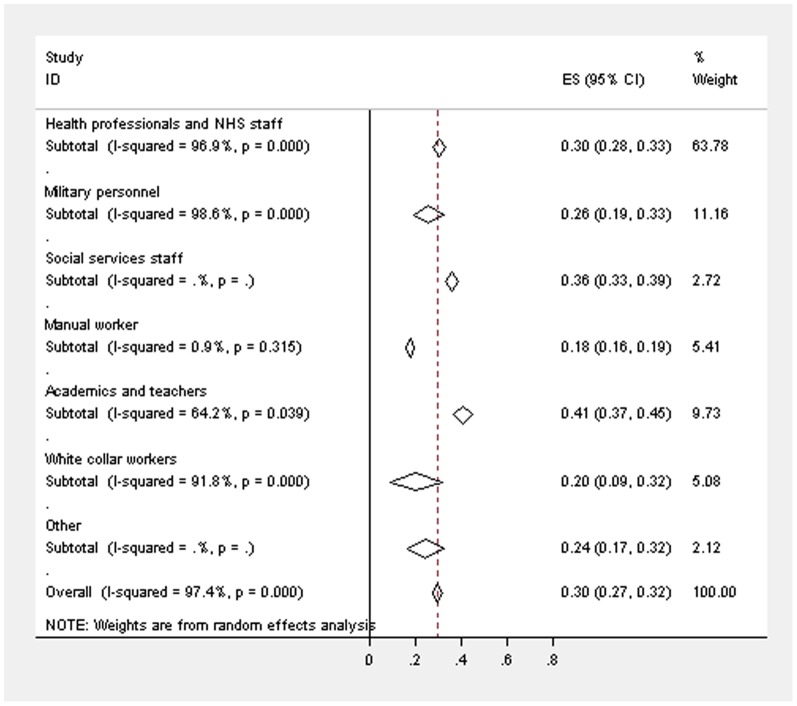
Forest plot displaying the weighted estimates for CMD caseness across the occupational groups (restricted to studies using GHQ-12 (3/4)).

There were 39 studies [2], [5], [11], [13], [14], [15], [16], [17], [18], [19], [20], [21], [22], [23], [24], [25], [26], [27], [28], [29], [30], [31], [32], [33], [34], [35], [36], [37], [38], [39], [40], [41], [42], [43], [44], [45], [46], [47], [48] which used the same version of the questionnaire and cut-off, yet heterogeneity between these studies was still high overall (I^2^ = 97.4%) and within the different occupational groups (academics and teachers - I^2^ = 64.2%; health professionals and NHS staff - I^2^ = 96.9%; manual workers - I^2^ = 0.9%; military personnel - I^2^ = 98.6%; white collar workers - I^2^ = 91.8%), even when exactly the same questionnaire was administered. The weighted prevalence estimate for CMD across all of these occupational studies was 29.6% (95% CIs 27.3–31.9%), with 40.9% (95% CIs 36.6–45.2%) for academics and teachers, 30.3% (95% CIs 27.6–33.0%) for health professionals, 17.6% (95% CIs 16.0–19.3%) for manual workers, 25.7% (95% CIs 19.0–32.5%) for military personnel, 24.3% (95% CIs 16.5%–32.2%) in a study of chaplains, 36.0% (95% CIs 33.1–38.9%) in one study of social services staff and 20.3% (95% CIs 8.7–31.8%) for white collar workers. Figure 3 displays the weighted estimates in these restricted studies.

#### Occupational studies which used the GHQ-12 (cut-off 3/4), random sampling and had a response rate ≥50%

In univariate meta regression analyses, neither the variable identifying studies as having conducted random sampling (β = 0.00, se[β] = 0.03, p = 0.93), nor that representing having a response rate ≥50% (β = −0.11, se[β] = 0.10, p = 0.28) were associated with the prevalence of CMD in the restricted occupational studies.

The meta-analysis restricted to the 10 studies [2], [5], [27], [33], [36], [37], [38], [39], [47], [48] which used the same version of the GHQ and the same cut-off, in addition to those which conducted random sampling, with a response rate of at least 50%, resulted in a lower overall prevalence estimate for CMD of 23.9% (95% CIs 20.5–27.4%), with less difference between the occupational groups which were represented in the studies that met these criteria: 21.5% (95% CIs 15.3–27.7%) for health professionals, 25.7% (95% CIs 19.0–32.5%) for military personnel, 24.3% (95% CIs 16.5%–32.2%) in a study of chaplains and 26.3% (95% CIs 21.1–31.5%) in a study of white collar workers.

### Population studies

#### Study characteristics

There were 13 population studies which had administered the GHQ, with the West of Scotland Twenty-07 study including 3 separate age cohorts, so there were 15 studies in total. The British Household Panel Survey, Health and Lifestyle Survey, MRC National Health and Development Study and the National Child Development Study all reported data from England, Scotland and Wales, with the English and Welsh Civil and Social Justice Survey only reporting data from two countries. The remaining studies included participants either from a single country (e.g. the Health Survey for England) or from a particular region in the UK (e.g. the Aberdeen Children of the 1950s Study) (table 2). All of the studies used random sampling or invited all participants in the sampling frame to participate in the study. Fourteen of the studies reported the response rate and 13 of these had a response rate of at least 50%.

**Table 2:** Data extraction table for population studies (ordered by name of study).

Name of the population study	Author and Year of publication/Name of Report	Population and location	Type of study (e.g. cohort study, panel study, cross sectional data collection)	Sampling frame and how participants were identified (give as much detail as is available)	Method of data collection (e.g. postal questionnaire, telephone interview, face-to-face interview)	No. of participants (no. who completed GHQ if different)	Response rate (and response at follow-up if the GHQ data is not from the first data collection)	Sample characteristics (gender, age)	Which version of the GHQ questionnaire was used (Cut-off used)	Prevalence CMD in full sample (%, 95% CI)
**Aberdeen Children of the 1950s Study (2001)**	Dundas et al. (2006)[95]	Adult follow-up of a survey of all children born in Aberdeen (1950–56) who attended a primary school in Aberdeen, Scotland in December 1962.	Adult follow-up of a cohort study	Survey of the total population of all children born in Aberdeen (1950–56) who attended a primary school in Aberdeen, Scotland in December 1962.	Postal questionnaire	11321 (7095)	Follow-up questionnaire received from 63%	47.7% males, 52.3% females. Mean age: 47 y.	GHQ-4 (0/1)	20.60%
**British Household Panel Survey (wave 1/1991)**	Weich, Lewis & Jenkins (2001)[96]	Individuals in private households in England, Wales and Scotland	First wave of panel survey	Representative sample of private households in England, Wales and Scotland. Two-stage stratified clustered probability design with postcode units as primary sampling unit.	Household interview	9612 (8191)	73.6% of households, 63% of individuals	Aged 16–75 y	GHQ-12 (2/3)	24.60%
**English and Welsh Civil and Social Justice Survey (2007)**	Balmer et al. (2010)[49]	Individuals living in private households across England and Wales	Cross sectional study	Stratified sample of 168 postcode sectors in England and Wales. The small user postcode address file was used as the sampling frame.	Household interview	3040	78% at household level. 59% of individuals.	46% males, 54% females. Age: 18–24 y -11.6%, 25–34 y - 14.7%, 35–44 y - 18.3%, 45–59 y - 24.8%, 60–74 y - 21.4%, 75 y+ - 9.2%.	GHQ-12 (3/4)	14.01%
**Health and Lifestyle Survey (1984/85)**	Lewis & Wilkinson (1993)[97]	Individuals living in private households in England, Wales and Scotland.	Cross-sectional population based survey	Random sample of people living in England, Wales & Scotland selected from the electoral register	Household interview. Participants given the GHQ-30 and asked to return it by post	12254 (6437)	52.5%	43.4% males, 56.6% females. Mean age 45.9 y.	GHQ-30 (4/5)	31.20%
**Health Survey for England (1995)**	Health Survey for England (1995) - main report [50]	Adults living in private households in England	Cross-sectional study	Representative sample of the population of England living in private households. A random sample of addresses selected from the postcode file, using a multi-stage sample design.	Computer assisted interview	16** **055	78% at household level. 73% of individuals.	45.7% males, 54.3% females	GHQ-12 (3/4)	17.3% overall (14% males, 20% females)
**MRC National Health and Development Study (53 y follow-up/1999)**	Hatch et al. (2009)[98]	Individuals born in England, Scotland and Wales, during one week in March 1946.	Cohort study - 53 year follow-up	Sample stratified by social class selected from all births in England, Scotland and Wales during one week in March 1946	Interview conducted by research nurse	3035 (2073)	83% of target sample, 57% of original cohort	50.7% males, 49.3% females.	GHQ-28 (5/6)	16.80%
**National Child Development Study (42 y follow-up/2000)**	National Child Development Study - data extracted from the dataset[51]	Individuals born in England, Scotland and Wales during 1 week of March 1958.	Cohort study - 42 year follow-up	All individuals born in England, Scotland and Wales in 1 week of March 1958 invited to participate	Household interview	11419 (11280)	71% of target sample	49.1% males, 50.9% females.	GHQ-12 (3/4)	18.80%
**Northern Ireland Health and Wellbeing Survey (1997)**	O'Reilly & Stevenson (2003)[52]	Households in Northern Ireland	Cross-sectional survey	Random sample of households in Northern Ireland, stratified by health board	Household interview, but the GHQ was self-completed	1694	75%	Aged 16–64 y	GHQ-12 (3/4) - The reported prevalence is weighted.	21.3% (need to enter this manually as weighted)
**Northern Ireland Household Panel Survey (wave 1/2001)**	Murphy & Lloyd (2007)[53]	Representative sample of private households in Northern Ireland	Cross-sectional study - wave 1 of a panel survey	Stratified random sampling. All members of a sampled household were interviewed.	Computer assisted personal interview	3163	Not stated	Not stated	GHQ-12 (3/4)	19.70%
**Renfrew and Paisley (MIDSPAN) study (1972–76)**	Pembroke et al. (2006)[99]	Adults aged 45–64 from two towns near Glasgow.	Cross-sectional study - first phase of a longitudinal study	All adults aged 45–64 from two towns near Glasgow from 1972–1976 were invited to participate.	Participants attended a clinic for interview	15411 (6575)	80% for the full sample	44.6% males, 55.4% females. Mean age 54 y.	GHQ-30 (3/4)	17.10%
**Scottish Health Survey (1995)**	Scottish Health Survey (1995) - main report [54]	Adults of ‘working age’ (16–64 years) living in private households in Scotland	Cross-sectional study	Nationally representative sample of the working age population of Scotland living in private households. Multi-stage stratified sample design, postcode primary unit, systematic selection of addresses, one person randomly selected per household using Kish Grid technique.	Computer assisted personal interview	7932 (7749)	81%	49% males, 51% females. Age range 16–64 y	GHQ-12 (3/4)	15.5% (12% men, 19% of women)
**West of London Survey (1977)**	Lewis & Wilkinson (1993)[97]	Random selection of adults selected from the electoral register in West London	Cross-sectional population based survey	Random selection from the electoral register in West London in 1977.	Household interview	8502 (5684)	66.9%	43.8% males, 56.2% females. Mean age 46 y.	GHQ-30 (1972 version) (4/5)	22.40%
**West of Scotland Twenty-07 Study - 1930s cohort (wave 1)**	Data provided by the Twenty-07 Study[55]	Stratified sample of three age cohorts of adults from the Central Clydeside Conurbation	Cohort study - first wave	Stratified sample (by unemployment and social economic group) of adults in the Central Clydeside Conurbation born around 1932	Household interview	1551 (1403)	48% of initial sample	Approximately 55 years old.	GHQ-12 (3/4)	17.60%
**West of Scotland Twenty-07 Study - 1950s cohort (wave 1)**	Data provided by the Twenty-07 Study[55]	Stratified sample of three age cohorts of adults from the Central Clydeside Conurbation	Cohort study - first wave	Stratified sample (by unemployment and social economic group) of adults in the Central Clydeside Conurbation born around 1952	Household interview	1444 (1233)	58% of initial sample	Approximately 35 years old.	GHQ-12 (3/4)	20.40%
**West of Scotland Twenty-07 Study - 1970s cohort (wave 2)**	Data provided by the Twenty-07 Study[55]	Stratified sample of three age cohorts of adults from the Central Clydeside Conurbation	Cohort study - second wave [participants were aged 15 years at wave 1]	Stratified sample (by unemployment and social economic group) of adults in the Central Clydeside Conurbation born around 1972	Household interview	1343 (1301)	60% of initial sample at wave 1 (84.7% participated at wave 2)	Approximately 18 years old	GHQ-12 (3/4)	28.70%

#### How was common mental disorder measured?

Eight of the 13 population studies administered the GHQ-12, with the most commonly used cut-off of 3/4. The follow-up to the Aberdeen Children of the 1950s study used a reduced 4-item version of the GHQ with a cut-off of 0/1. The MRC National Health and Development Study used the GHQ-28 (5/6) and the remaining three studies used the GHQ-30 (two with a 4/5 cut-off and the Renfrew & Paisley study with a particularly low 3/4 cut-off).

#### Prevalence of common mental disorder

**Figure 4:**
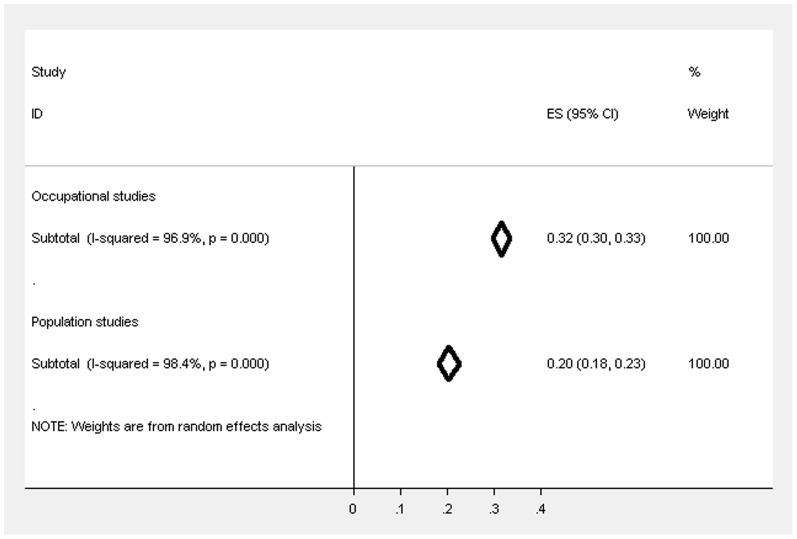
Forest plot displaying the weighted estimates for CMD caseness between the occupational and population studies.

Figure 4 displays the weighted estimates for CMD caseness for the population and occupational studies, showing that the estimate was 20.4% (95% CIs 18.2–22.6%) for population studies and there was high heterogeneity (I^2^ = 98.4%). The prevalence across the studies ranged from 14.0% scoring above the cut-off for symptoms of CMD in the English and Welsh Civil and Social Justice Survey to 31.2% in the Health and Lifestyle Survey.

#### Population studies which used the GHQ-12 (cut-off 3/4)

**Figure 5:**
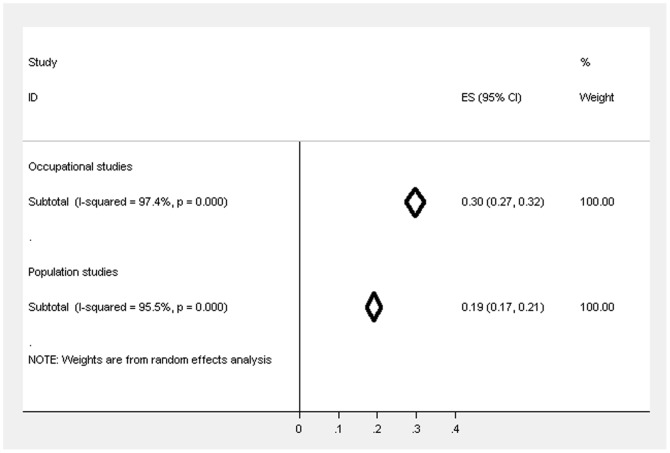
Forest plot displaying the weighted estimates for CMD caseness between the occupational and population studies (restricted to studies using GHQ-12 (3/4)).

There were 9 studies [49], [50], [51], [52], [53], [54], [55] that used the same questionnaire and cut-off and there was high heterogeneity between these studies (I^2^ = 95.5%). The weighted prevalence estimate (see figure 5) across these studies was 19.1% (95% CIs 17.3–20.8%).

#### Population studies which used the GHQ-12 (cut-off 3/4), random sampling and had a response rate ≥50%

In a univariate meta regression analysis, having a response rate ≥50% (β = −0.06, se[β] = 0.16, p = 0.72) was not associated with the prevalence of CMD in the restricted population studies. The meta-analysis restricted to the 7 studies [49], [50], [51], [52], [54], [55]which used the same version of the GHQ and the same cut-off, in addition to those which conducted random sampling, with a response rate of at least 50%, reported a prevalence estimate of CMD of 19.2% (95% CIs 17.1–21.3%), with evidence for high heterogeneity between these restricted studies (I^2^ = 96.4%).

### Comparing the prevalence of CMD in occupational and population studies

**Figure 6:**
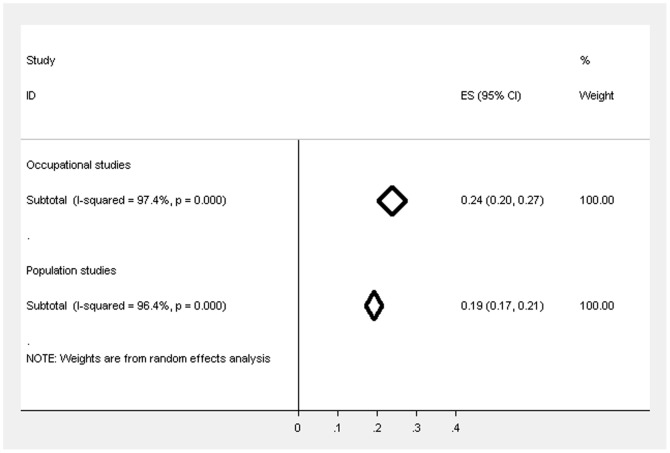
Forest plot displaying the weighted estimates for CMD caseness between the occupational and population studies (restricted to studies using GHQ-12 (3/4), response rate ≥50% & random sampling).

Figure 4 highlights the trend for the weighted estimates of CMD to be higher overall in occupational studies, regardless of the occupational group, compared to the population studies and the confidence intervals do not overlap suggesting that the difference is statistically significant. When restricted to studies that used GHQ-12 (cut-off 3/4) the prevalence estimates (see figure 5) also suggest that the prevalence is significantly higher in occupational studies compared to population studies, but the differences is reduced and no longer significant after further restricting by response rate and sampling method (see figure 6; occupational studies −23.9%, 95% CIs 20.5%–27.4%; population studies −19.2%, 95 CIs 17.1%–21.3%).

## Discussion

The main finding of this systematic review is that people appear less likely to report symptoms of CMD in the context of a population based study rather than in a study of the occupational group to which they belong. This difference was reduced but not fully accounted for by differences between these types of studies, in relation to the quality of the studies. This effect is unlikely to reflect true differences in the level of CMD symptoms experienced, because population studies aim to comprise all sections of the population, including those with chronic health problems, long term disabilities and the unemployed, whilst occupational studies will be subject to the health worker effect [7]; so one would anticipate the reverse. There are aspects of work which are associated with increased psychological distress (e.g. high demands and low decision latitude [56]), however, the benefits of employment, over not working, for mental health have consistently been outlined and it is established that the prevalence of CMD is lower in working populations compared to the general population [57]. There were also unexpected findings within the occupational groups: occupations for which one would expect a higher level of traumatic events, such as the military and police, were not found to have a higher prevalence of CMD than other occupational groups, including academics, teachers, white collar workers and social services staff.

We propose that studies directed at the mental health or “stress levels” of particular occupations may be subjected to a systematic bias, one that is not present in true population studies when participants are not selected purely because they belong to a specific occupation. Numerous occupational studies are actually labelled as studies of “work stress”, which may give rise to a framing effect. There is evidence for strong contextual effects in previous experimental and observational studies [58], [59], [60], [61], which can be defined as the effect of environmental factors on subjective outcomes, in addition to bias in self-report psychological measures [62]. The setting in which a questionnaire is completed is likely to influence responses. Responses in the occupational studies may have been biased by a framing effect [63], with the emphasis on job related questions potentially leading to individuals venting their work frustrations through questionnaires which provide an opportunity to report dissatisfaction. This framing effect may be heightened, or conversely lessened, depending on where the GHQ is embedded within the questionnaire and its positioning relative to other psychosocial measures.

Within the occupational groups certain other trends also became apparent, before any restrictions had been made to the meta-analyses. This study was constrained to published research studies (so we did not have data on all occupational groups in the UK), but the overall findings suggested that teachers and academics (with this category predominantly comprising studies of university academics) and social services staff had higher levels of CMD (although the differences were not statistically significant). There is further evidence for higher rates of CMD in teachers in other studies. Analyses of the 2000 UK Adult Psychiatric Morbidity Survey showed that the prevalence of psychological disorder was highest in managers & administrators, teaching professionals, other associate professionals, clerical and secretarial, ‘other’ sales and personal service occupations. However the prevalence of psychological disorder was higher in primary and secondary teachers, compared to higher education teachers [64], so is not completely in agreement with this review which predominantly included university academics. A further study examining sickness absence from 2001 to 2007 in the Netherlands showed that the percentage of sick days due to CMD was highest in the education sector, followed by financial services and health care sectors [65]. The Adult Psychiatric Morbidity Survey is a population study and the latter study measured actual behaviour in response to CMD, suggesting that the difference between occupational groups is unlikely to be a consequence of contextual effects which seem to be stable across occupations.

A surprising finding within this review was the variation in prevalence estimates for CMD caseness between the population studies, even when restricted to those which used the same questionnaire and threshold. The population studies identified within this review are often used as a population reference for measures of mental health [66], but this study shows that the comparison may differ depending on which population study is chosen. Whilst both the British Household Panel Survey and Health Survey for England are considered to recruit representative samples, there is still a difference between the prevalence of CMD caseness for these surveys, indicating that this either results from a sampling effect or from the sensitivity of the GHQ to other factors which may differ between surveys. These include the position of the GHQ within the overall questionnaire, the length of a questionnaire and finally whether it was self-completion or interview administered.

### Strengths and weaknesses

We have conducted a comprehensive review of all papers published since 1990 that have administered the GHQ, within the two areas of research in which this questionnaire is most commonly used, both occupational and population research. This is the first study of which we are aware to compare occupational and population rates of CMD, that is not restricted to a particular occupation. Weaknesses of this review include the fact that the majority of occupational studies of mental health have been conducted within health professionals, most likely due to the salience of these issues to the authors, and there were fewer studies in occupations that are considered to be at a high risk of stress, such as the police. The version of the GHQ administered differed between studies, in addition to the cut-off that was applied. Whilst we tried to control for this by restricting some of the analyses to comparable studies, this is a general limitation of comparisons between GHQ studies which is not always taken into account by study authors. However, the findings do not suggest that the studies with the highest prevalence were those using the lowest cut-off. Further limitations include the sampling differences between the population studies and the difference in location both within the population studies and between the population and the occupational studies. The majority of the occupational studies were conducted in a specific area as opposed to being national studies of a particular occupation. There was also evidence that in general the population based studies were of a higher quality than occupational studies, and the difference between them was reduced after making restrictions to the meta-analysis based on study quality. Finally, there was very high heterogeneity in the meta analyses of both the occupational and population data, which remained when the studies included in the meta-analysis were restricted to be more comparable [67], [68].

### Implications

The primary implication of this research relates to the sensitivity of the GHQ, which asks about ‘recent’ symptoms of mental health, to factors other than objective mental health and to the potential framing effect resulting from the overall narrative of a questionnaire or interview. However, we suggest that other self-reported measures of mental health may be subject to contextual effects, and that interpreting the results of any single study without considering the context in which it was given, and the possible bias that introduces, may lead to flawed conclusions. Hence for example, if an individual reports higher levels of psychological symptoms within the context of an occupational study, this may be a reflection of dissatisfaction with their job as opposed to reflecting depression or unhappiness with their life outside of work. These types of effects have previously been shown in individual studies, but we have systematically reviewed evidence across a range of studies. The elevated levels of CMD evidenced in occupational studies may be reduced if mental health is assessed separately to job satisfaction and other occupational constructs and thought should be given as to where the mental health measures are incorporated in a questionnaire.

## Conclusions

This review has shown that more individuals scored above the threshold for CMD caseness in occupational studies, compared to population studies, even when accounting for the version of the questionnaire that was administered and the threshold used to classify CMD. We propose that this difference may have resulted from the context of the occupational studies resulting in higher reports of psychological symptoms, in addition to differences between these types of studies in study quality.

## Supporting Information

Checklist S1 PRISMA Checklist.
(DOCX)
